# Dioscin Alleviates Cardiac Dysfunction in Acute Myocardial Infarction *via* Rescuing Mitochondrial Malfunction

**DOI:** 10.3389/fcvm.2022.783426

**Published:** 2022-03-04

**Authors:** Tianyu Shen, Dayin Lyu, Mengping Zhang, Hui Shang, Qiulun Lu

**Affiliations:** ^1^Department of Orthopaedic, Taihe Hospital, Hubei University of Medicine, Shiyan, China; ^2^Key Laboratory of Cardiovascular and Cerebrovascular Medicine, Collaborative Innovation Center for Cardiovascular Disease Translational Medicine, Nanjing Medical University, Nanjing, China; ^3^Department of Hematology, Taihe Hospital, Hubei University of Medicine, Shiyan, China

**Keywords:** myocardial infarction, dioscin, mitochondrial dysfunction, ROS, KREB'S cycle

## Abstract

Myocardial infarction is one of the most severe heart diseases, leading to sudden death. Currently, angiography and stenting are widely performed in clinics, yet more effective treatment is still needed. Herein, we presented that dioscin, a natural product, showed protective effect on infarcted hearts *via* mitochondrial maintenance. Upon dioscin treatment, cardiac dysfunction was alleviated, and remodeling is prevented. Mechanistically, disocin maintains mitochondria function through the maintenance of Kreb's cycle, and suppresion of ROS accumulation. In this way, by targeting mitochondrial dysfunction, dioscin is a potential drug for infarcted hearts.

## Introduction

Nowadays, it is widely considered that cardiovascular diseases are one of the major causes for human mortality around the world (1). Particularly, myocardial infarction (MI) affects large population in both developing and developed countries (2). It characterized with dysfunction of energy supply and reactive oxygen species (ROS) generation relates to mitochondrial-based disorder, which leads to pathogenesis of MI and even heart failure (3). Therefore, novel drugs of protecting the heart against myocardial infarction are urgently needed.

Mitochondrial dysfunction is associated with the pathogenesis of myocardial infarction (4). Mitochondrial-derived ROS represent a major source of oxidative stress in cardiomyocytes (5). On the other hand, the primary source of energy generating processes is located at mitochondria among the cell, which is severe sensitive to free radical occurrence in cardiomyocytes (6). Taken together, mitochondria maintenance would moderate heart injury after infarction.

Recently, researches indicated that natural productions from some herbals have potential therapeutic effect for the prevention and treatment of cardiovascular diseases (7). The protective effects are associated with mitochondrial function, including alleviating mitochondrial oxidative stress, de-activation of respiratory chain enzymes, postponing mitochondrial Kreb's cycle, and maintenance of mitochondrial membrane potential (Δψ) and ATP content (8–10). Dioscin, a natural steroid saponin isolated from the root bark of wild *dioscorea nipponica*, is reported to treat MI by promoting angiogenesis (11). Additionally, it was reported that dioscin maintains mitochondrial dynamics, which rescues silicosis by reducing mitochondrial-dependent apoptosis (12). However, whether dioscin protects cardiac injury against MI by rescuing mitochondrial dysfunction remains unclear.

Here, our work revealed that dioscin attenuates cardiac dysfunction induced by myocardial infarction. Administration of dioscin benefits infarcted hearts could significantly enhance mitochondrial Kreb's cycle and activities of respiratory chain enzymes, reduce ROS by promoting the antioxidant enzyme activities, and suppress the cardiomyocytes apoptosis by rescuing mitochondrial member potential. Taken together, our results showed that dioscin could alleviate MI *via* attenuate mitochondrial malfunction.

## Materials and Methods

### Animal Model and Chemicals

Mice were male and at age of 8 weeks old. All male C57BL/6 were from Jiangsu Animal Experimental for Medical and Pharmaceutical Research Center. The *in vivo* experiments were approval of Institutional Animal Care and Use Committees at the University of Nanjing Medical University (Number: 1912034) and carried out in accordance with the guidelines. Mice were kept with 12 h light/12 h dark cycle at 23 ± 3°C and 30–70% humidity.

Infarction mouse model was generated through ligature of the left anterior descending (LAD) as described previously with male mice (11). Briefly, mice were anesthetized with 1.5–2% isoflurane, and then a left thoracotomy and pericardiotomy was performed. The left anterior descending was completely occluded by a knot with 7–0 non-absorbable surgical suture. After the ribs and skin were closed with 5–0 silk, the mouse remained under anesthesia until succinylcholine was completely metabolized. For the procedure of sham surgery, mice underwent the same procedure without the ligation of the artery.

Dioscin (80 mg/kg/day, Di'ao group, Chengdu, China) was administrated with mice for 2 weeks beginning on the day of operation. Briefly, the dioscin was first dissolved in dimethyl sulphoxide (DMSO) and then added 5% sodium carboxymethyl cellulose (CMC-Na) to make the volume ratio for 1:19. Consequently, the mice were given the dissolution by intragastric administration for continuous 14 days after myocardial infarction 1 day. The treatment groups were given dioscin solution and the control groups were given same volume of 5% CMC-Na vehicle solution. The preparation and application of the drug's dissolution should be completed in 1 day.

### Echocardiography

Transthoracic echocardiographic analysis was performed 2 weeks after the sham or MI surgery as previously described (13). Echocardiography on unconscious mice was used with a Vevo 2100 system (VisualSonics). The echocardiography was blinded to the mice in this study.

### Histological Analysis

Hematoxylin and eosin and Masson trichrome staining were performed as described previously (14). The whole hearts were collected and fixed in 4% paraformaldehyde, embedded in paraffin, and then cut into 5-μm serial sections. Image analysis and quantification of fibrosis was performed by a blinded investigator using Image J software.

### Mitochondrial Isolation

The purification of cardiac mitochondrial fraction from the heart tissues or cultured primary cardiomyocytes was followed as previously described (15), using Mitochondrial Extraction Kit (Solarbio Life Sciences, SM0020) according to the manufacturer's instructions.

### Estimation of Reactive Oxygen Species

Dihydroethidium (DHE) staining (Beyotime, China) was performed as described previously (16). Briefly, heart samples were embedded in OCT compound and transverse sectioned at 5 μm thickness to perform staining and then conserved at −80°C. Next, heart sections were set in room temperature for 10 min and then washing by PBS three times for 5 min. Further, the sections were incubated with the DHE (1:1,000) for 30 min in wet box at 37°C. Finally, after washing by PBS three times for 5 min, co-stain with DAPI. The imagines are photographed by confocal, and we use the same intensity of laser.

### Estimation of Mitochondrial Enzymatic and Non-enzymatic Antioxidant

The activity of glutathione peroxidase (GPx), superoxide dismutase (SOD), glutathione-S-transferase (GST), catalase (CAT), glutathione (GSH) was determined in cardiac tissues, using Micro Superoxide Dismutase (SOD) Assay Kit (Solarbio Life Sciences, BC0175, BC0205, BC1195, BC0355, and BC1175) according to the manufacturer's instructions.

### TCA Cycle Enzyme Activities and Respiratory Chain Enzymes

Effect of dioscin on MI-induced mitochondrial damage was measured by detection of the activities of mitochondrial enzymes such as malate dehydrogenase (MDH), isocitrate dehydrogenase (ICDH), and succinate dehydrogenase (SDH) using Micro Isocitrate Dehydrogenase Mitochondrial (ICDHm) Assay Kit (Solarbio Life Sciences, BC2165), Micro Succinate Dehydrogenase (SDH) Assay Kit (Solarbio Life Sciences, BC0955) and Micro NADP-Malate Dehydrogenase (NAD-MDH) Assay Kit (Solarbio Life Sciences, BC1055) according to the manufacturer's instructions.

The nicotinamide adenine dinucleotide dehydrogenase (NADH) activity was determined in cardiac tissues as reported (17), using Micro Nicotinamide Adenine Dinucleotide Dehydrogenase (NADH) Assay Kit (Solarbio Life Sciences, BC0635) according to the manufacturer's instructions.

The cytochrome c-oxidase (NCR) activity was determined in cardiac tissues, using Micro NADPH-cytochrome C Reductase Assay Kit (Solarbio Life Sciences, BC2725) according to the manufacturer's instructions.

### Cell Culture

H9C2 cells were cultured in DMEM (Servicebio) medium with 10% FBS (Gibco, Life Technologies) and 1% penicillin/streptomycin (Beyotime). Final concentration of 100 μM CoCl_2_ (Sigma-Aldrich) was added to simulate hypoxia condition. After 24 h of treatment, the extraction of mRNA was performed. And after 48 h, the corresponding staining was performed.

### qRT-PCR

Total RNA was extracted using RNeasy Mini Kit (Qiagen). cDNA was generated using HiScript III 1st Strand cDNA Synthesis (Vazyme) and then qPCR was performed using on QuantStudio 5 applied biosystems (Thermo Fisher Scientific) with Hifair III One Step RT-qPCR SYBR Green Kit (Yeasen), all according to the manufacturer's instructions. Actin was regard as control and the data analysis was using 2CT. Obtaining form three independent experiments, and each sample was repeated duplicated in each experiment. Primer sequence are listed below.

For mouse qPCR:

*Anp*: TTCTTCCTCGTCTTGGCCTTT, GACCTCATCTTCTACCGGCATCT;

*Bnp*: CACCGCTGGGAGGTCACT, GTGAGGCCTTGGTCCTTCAAGGTCACT;

β*-mhc*: ATGTGCCGGACCTTGGAA, CCTCGGGTTAGCTGAGAGATCA;

*Bax*: CCCGAGAGGTCTTTTTCC, GCCTTGAGCACCAGTTTG;

*Bcl*_2_: CCTGGCTGTCTCTGAAGACC, CTCACTTGTGGCCCAGGTAT;

*Actin*: CATCGTCCACCGCAAATG, CACCTTCACCGTTCCAGTT.

### Mitochondrial Membrane Potential

Mitochondrial membrane potential was measured by the method of fluorescent probe, using the MitoTracker® Green FM (Yeasen, 40742ES50) according to the manufacturer's instructions.

### TUNEL Staining

TUNEL staining was performed as previously report (18). The suitable density H9C2 cells were induced to CoCl_2_ (100 uM, Sigma, 449776) under the condition of treatment with dioscin (5 mg/L) for 48 h, compared to control group. Further, the H9C2 cells were fixed in 4% paraformaldehyde and stained with One Step TUNEL Apoptosis Assay Kit (Beyotime, C1086). TUNEL positive were counted from different images per sample, and the averages were presented.

### Estimation of Caspase-1 Activities

The cysteine-requiring aspartate protease 1 (Caspase-1) activity was determined in cardiac tissues, using Caspase-1 Reductase Assay Kit (Hypertime, China) according to the manufacturer's instructions.

### Statistical Analysis

Data were presented as mean ± standard error of the mean (SEM) using GraphPad Prism 8. Two-tailed unpaired *t*-test were used to determine the differences between two groups, and two-ANOVA was employed for comparisons of the differences between multiple groups. Statistical significance is indicated by ^*^ *p* < 0.05.

## Results

### Dioscin Alleviates the Mitochondrial Impairment by CoCl_2_ in H9C2 Cells

**Figure 1:**
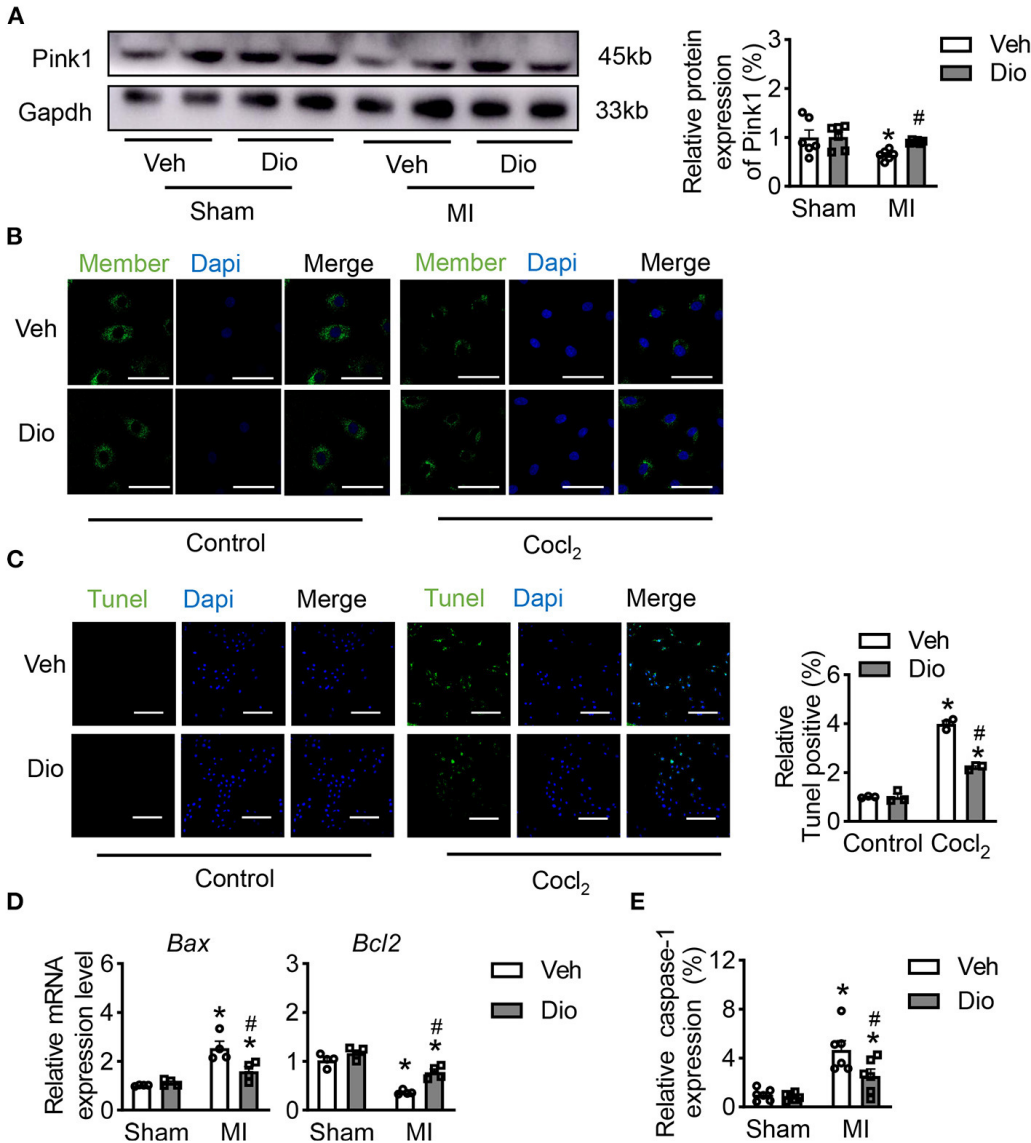
Dioscin alleviates the mitochondrial injury by CoCl2 in H9C2 cells. **(A)** The protein level of Pink1 in heart tissue (*n* = 6). **(B)** Representative images of the active mitochondrial, bar = 20 μm (*n* = 3). **(C)** Representative images of TUNEL staining to detect the anti-apoptosis role of dioscin *in vitro*, bar = 50 μm (*n* = 3). **(D)** The mRNA level of *Bax* and *Bcl2 in vivo* (*n* = 4). **(E)** The measurable of caspase-1 *in vivo* (*n* = 6). Data are mean ± SEM. *P < 0.05 vs. sham group, ^#^*P* < 0.05 vs. MI group.

Mitochondrial dysfunction is one of the primary causes for cardiac death in response to infarction. Firstly, we performed *in vivo* experiment to assess the mitochondrial injury after dioscin treatment in MI heart tissues. The protein level of Pink1 revealed that dioscin recovered the expression of Pink1 after MI, indicating dioscin has a protective role in mitochondria (Figure 1A). Then, the protective effect of dioscin was evaluated on mitochondrial membrane potential. The disturbance of the mitochondrial membrane potential (MMP) caused by CoCl_2_, and this injury was mitigated by dioscin (Figure 1B). Considering the impaired mitochondria could lead to cell death, the dead cells were detected by using TUNEL assay. This showed that dioscin recovered the numbers of H9C2 cells in response to hypoxic condition (Figure 1C). Additionally, the mRNA levels of *Bax* and *Bcl*_2_ as apoptotic markers were detected in hypoxic H9C2 cells after dioscin treatment. The results showed that dioscin exerts anti-apoptotic effects and prevents cells from death by downregulating *Bax* expression and increasing *Bcl*_2_ expression (Figure 1D). Furthermore, the activities of cysteine-requiring aspartate protease 1 (caspase-1) was repressed in hypoxic cells after dioscin incubation, suggesting that dioscin repressed apoptosis caused by hypoxia (Figure 1E). These data suggest that dioscin plays protective roles against hypoxia induced mitochondrial injury to mitigate cellular apoptosis.

### Dioscin Protects Cardiac Dysfunction Against Myocardial Infarction

**Figure 2:**
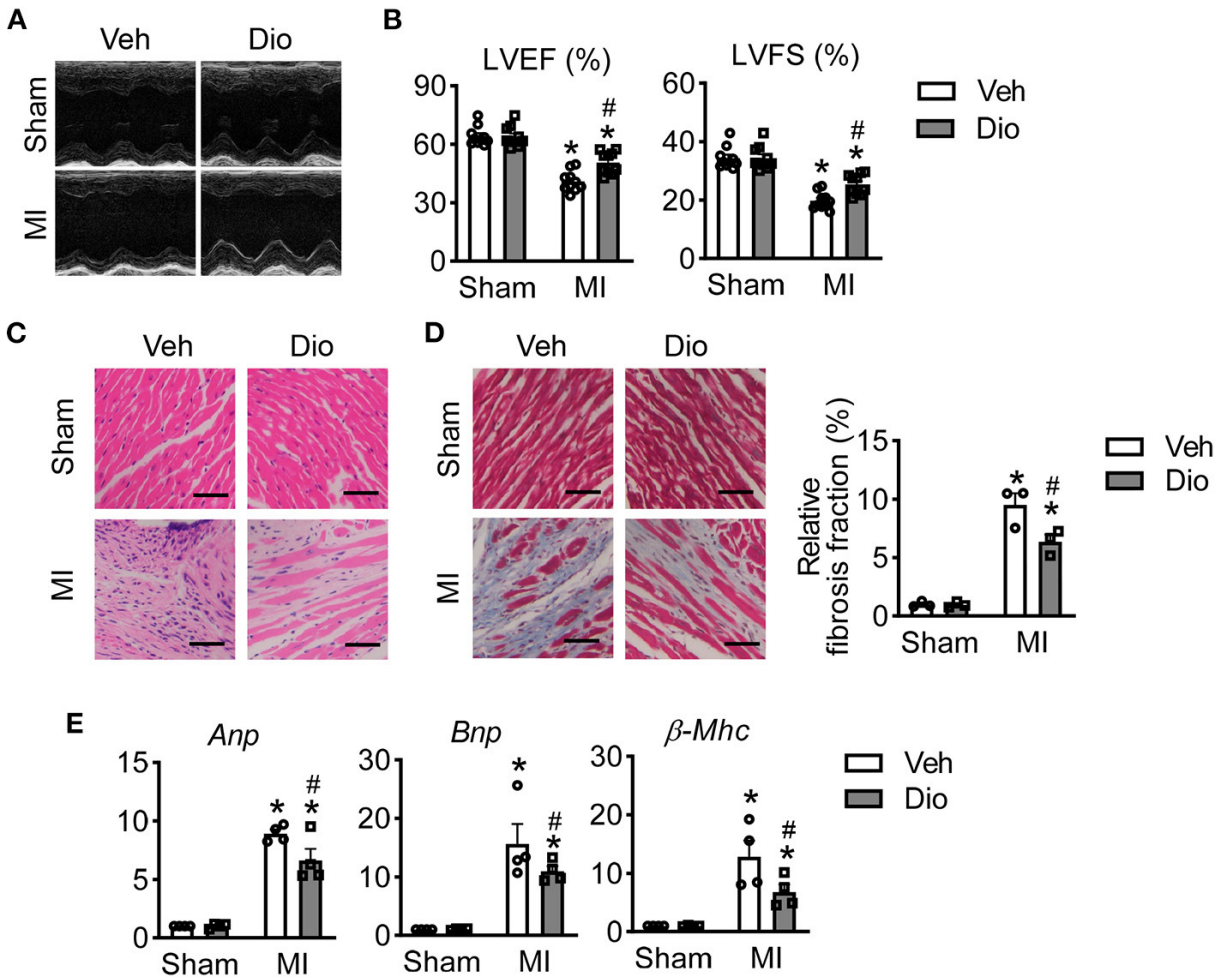
Dioscin protects mice against infarcted injury. **(A)** Representative images of echocardiographic measurement (*n* = 10). **(B)** Echocardiographic parameters for left ventricular ejection fraction (LVEF, %) and left ventricular fractional shortening (LVFS, %) (*n* = 10). **(C)** Representative images of HE staining, bar = 100 μm (*n* = 3). **(D)** Representative images of Masson trichrome stained and its quantification, bar = 100 μm (*n* = 3). **(E)** The mRNA level of heart failure markers for natriuretic peptide A (*Anp*), natriuretic peptide B (*Bnp*) and beta-myosin heavy chain (β*-Mhc*) (*n* = 4). Data are mean ± SEM. *P < 0.05 *vs*. sham group, ^#^*P* < 0.05 *vs*. MI group.

To determine the therapeutic role of dioscin *in vivo*, mice were subjected to MI surgery. Two weeks after dioscin administration, the echocardiography was performed to monitor cardiac function. Representative echocardiograph showed that the infarcted mice exhibited left chamber dilation, and dioscin administration alleviated the cardiac deformation (Figure 2A). Then, cardiac function analysis showed that the left ventricular ejection fraction (LVEF) and left ventricular fractional shortening (LVFS) were recovered after administration of dioscin in infarcted mice (Figure 2B). Additionally, haematoxylin and eosin (HE) staining of heart sections from mice in each group showed that the normal arrangement of cells was destroyed in infarcted heart increased, which could be prevented by dioscin (Figure 2C). Representative images exhibited that less collagen deposition after dioscin treatment by the size of blue region compared to MI group, which was decreased about 50% (Figure 2D), Suggesting dioscin alleviated cardiac fibrosis in infarcted hearts. Additionally, the expression level of heart failure markers was detected in hearts, including natriuretic peptide A (*Anp*), natriuretic peptide B (*Bnp*) and beta-myosin heavy chain (β*-Mhc*). It showed that the increased levels of *Anp, Bnp* and β*-Mhc* mRNA levels in heart tissues from infarcted mice were suppressed by dioscin administration (Figure 2E). These results indicate that dioscin administration preserved the cardiac function by alleviating cardiac fibrosis after MI.

### Dioscin Prevents Cardiac Remodeling in MI Mouse Model

**Figure 3:**
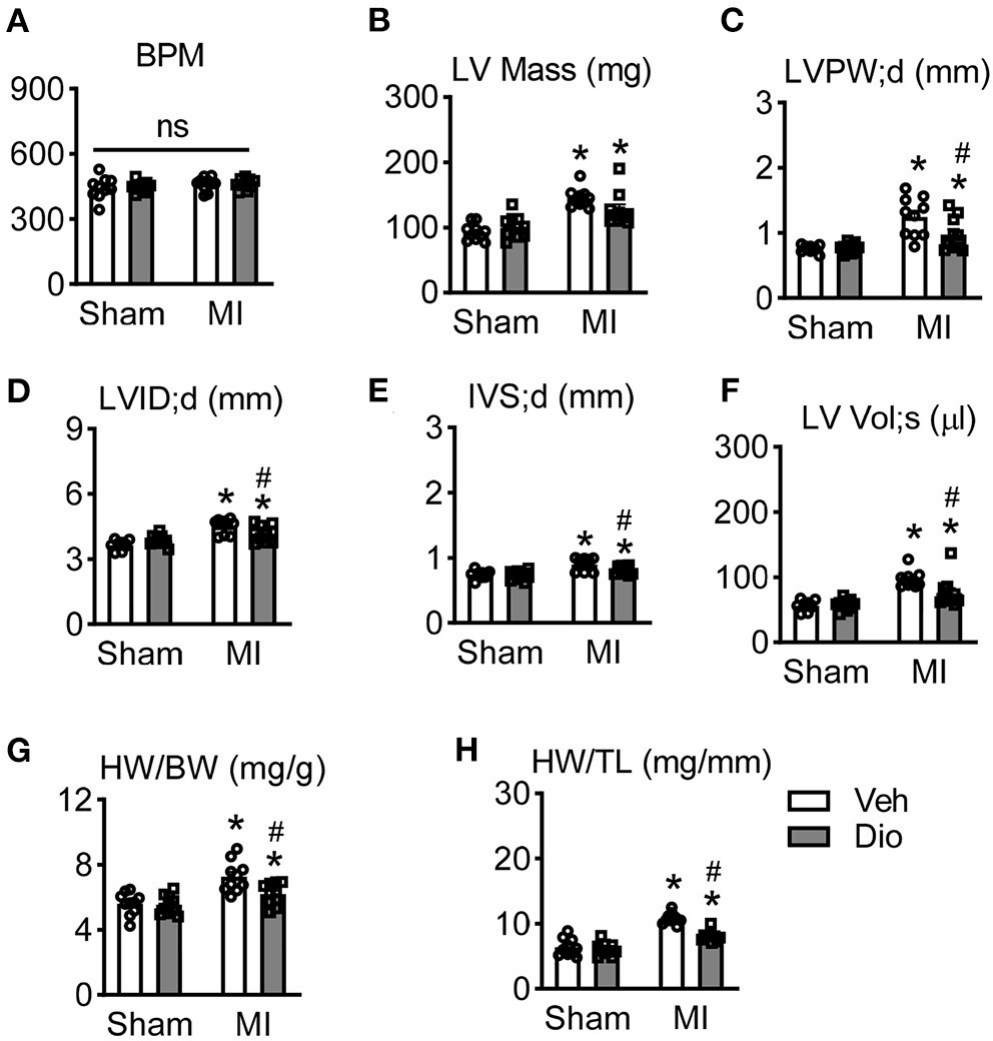
Dioscin prevents cardiac remodeling in response to myocardial infarction. **(A)** The analysis of beat per minute (BPM). Echocardiographic parameters of **(B)** left ventricular mass (LV Mass; mg), **(C)** end-diastolic left ventricular posterior wall (LVPW; d, mm), **(D)** end-diastolic left ventricular inner diameter (LVID; d, mm), **(E)** end-diastolic left ventricular septum (IVS; d, mm), and **(F)** left ventricular end-systolic volume (LV Vol; s, μl). **(G)** The ratio of heart weight to body weight (HW/BW; mg/g). **(H)** The ratio of heart weight to tibia length (HW/TL; mg/mm). *n* = 10 each group. Data are mean ± SEM. *P < 0.05 *vs*. sham group, ^#^*P* < 0.05 *vs*. MI group.

Considered the recovery of cardiac function occurs in infarcted hearts after disocin administration, cardiac remodeling was measured. There is no significant difference of heart rate among these four groups (Figure 3A). It was consistent with previous report that infarction increases left ventricular mass. Such increase was significantly repressed after administration of dioscin (Figure 3B). Other cardiac structure parameters, such as left ventricular posterior wall end-diastolic thickness (LVPW; d), left ventricular inner diameter end-diastolic thickness (LVID; d), left ventricular septum (IVS), and left ventricular end-systolic volume (LV Vol; d) were also sufficiently persevered after dioscin administration in infarcted hearts (Figures 3C–F). Additionally, mice subjected to MI showed a significant increase in heart weight/body weight (HW/BW) ratio and heart weight/tibia length (HW/TL), which could be ameliorated by dioscin e (Figures 3G,H). These results demonstrate that dioscin attenuated MI-induced heart remodeling.

### Effect of Dioscin on the Activities of Mitochondrial Kreb's Cycle in Heart After MI

**Figure 4:**
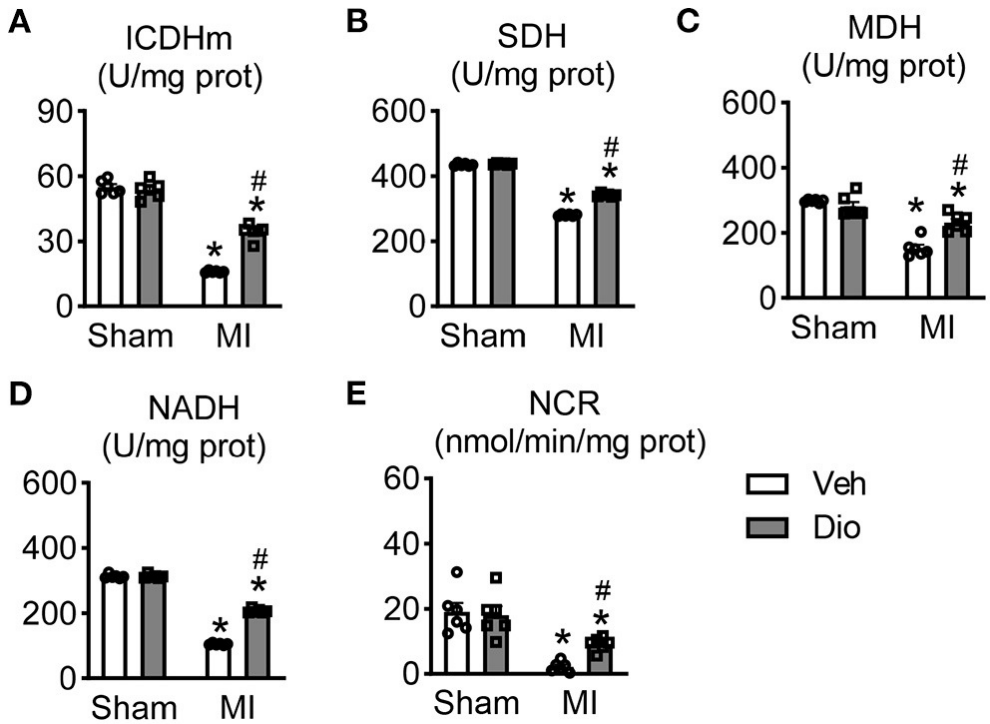
Effect of dioscin on the activities of mitochondrial KREB cycle and respiratory chain enzymes in hearts after MI. The key enzymes of tricarboxylic acid cycle (TCA) of **(A)** isocitrate dehydrogenase (ICDH; U/mg prot), **(B)** succinate dehydrogenase (SDH; U/mg prot), and **(C)** malate dehydrogenase (MDH; U/mg prot). The measureable of respiratory chain enzymes of **(D)** nitotinamide adenine dinucleotide dehydrogenase (the complex I, NADH; U/mg prot) and **(E)** cytochrome c-oxidase (the complex III, NCR; nmol/min/mg prot). *n* = 6. Data are mean ± SEM. *P < 0.05 *vs*. sham group, ^#^*P* < 0.05 *vs*. MI group.

The impaired function of creatine kinase and decreased mitochondrial respiration are often observed in myocardium after pathological injury, including myocardial infarction and hypertrophy (19, 20). In attempt to explore the effect of dioscin in the process of energy metabolism, the activities of mitochondrial enzymes were measured in mice hearts. The activities of isocitrate dehydrogenase (ICDH), succinate dehydrogenase (SDH) and malate dehydrogenase (MDH) in mitochondria were significantly downregulated in the infarcted hearts compared to the sham group (Figures 4A–C). The activities of these enzymes were remarkable enhanced by treatment with dioscin in MI group (Figures 4A–C). These proved the beneficial role of dioscin on Kreb's cycle enzymes, which are the major source of ATP production in the cells. We further examined the activities of respiratory chain enzyme responsible for the synthesis of ATP. The activities of nicotinamide adenine dinucleotide dehydrogenase (NADH) (the Complex I) and cytochrome c-oxidase (the Complex III) were significantly reduced in the mitochondrial isolated from cardiac tissues obtained from the mice subjected to MI. Conversely, the decline was inhibited by treatment with dioscin (Figures 4D,E). Overall, these findings suggest that dioscin promotes the activities of mitochondrial Kreb's cycle in myocardial infarction.

### Dioscin Modulates Mitochondrial Antioxidant Status in Heart

**Figure 5:**
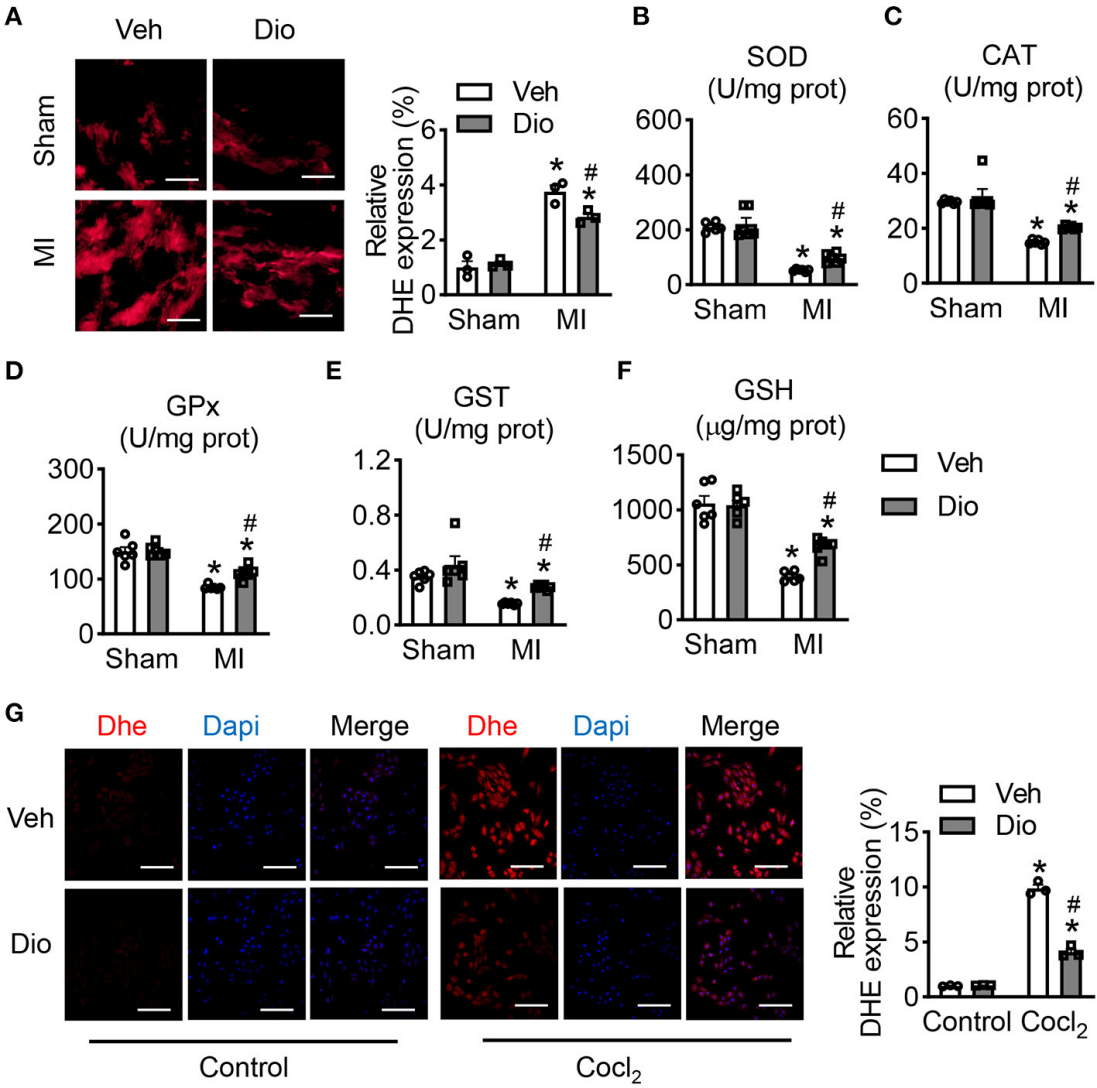
Dioscin modulates mitochondrial antioxidant status. **(A)** Representative images of DHE in heart section and its qualification, bar = 50 μm (*n* = 3). The antioxidant enzymes of **(B)** superoxide dismutase (SOD; U/mg prot), **(C)** catalase (CAT; U/mg prot), **(D)** peroxidase (GPx; U/mg prot), **(E)** glutathione-S-transferase (GST; U/mg prot), and **(F)** glutathione (GSH; U/mg prot) (*n* = 6). **(G)** Representative images of DHE in H9C2 cells and its quantification, bar = 50 μm (*n* = 3). Data are mean ± SEM. *P < 0.05 *vs*. sham group, ^#^*P* < 0.05 *vs*. MI group.

In the infarcted hearts, subcellular organelles undergo stress caused by the released of reactive oxygen species (ROS) from mitochondria. Hence, prevention of ROS accumulation in the subcellular compartments are essential for the restoration of the normal cardiomyocyte function. Representative images of dihydroethidium (DHE) staining showed that dioscin attenuated the increase of ROS in response to MI surgery (Figure 5A). We further detected the expression of antioxidant enzymes in mitochondria. Consistently, the activities of superoxide dismutase (SOD), catalase (CAT), glutathione peroxidase (GPx) and glutathione-S-transferase (GST) were declined in the animals subjected to MI, but recovred after dioscin treatment (Figures 5B–E). The decrease in the endogenous antioxidant defense enzyme system was corroborated to a diminished glutathione (GSH) content (Figure 5F). Similarly, the ROS accumulation was measured in H9C2 cells. Consistent with *in vivo* experiments, dioscin suppressed the enhancement of ROS caused by CoCl_2_ (Figure 5G). These results confirmed that dioscin abrogated the oxidative stress, enhancing the endogenous antioxidant defense system. Taken together, our study demonstrates that dioscin protects heart dysfunction against myocardial infarction by suppressing the oxidative stress.

## Discussion

In this study, the activities of antioxidant enzymes were obviously enhanced by treatment of dioscin to further eliminate the production of ROS. Herein, dioscin alleviated cardiac dysfunction in mouse model of MI *via* mitochondrial protection. In detail, dioscin speeds up the Kreb's cycle by up-regulating the activities of enzymes in mitochondrial. Our study further confirmed the therapeutic effect of dioscin in mice infarcted/hypoxic modelshearts and provided the promising administration of dioscin for heart diseases in clinics.

It has been reported that the mitochondrial ROS could be an index of oxidative stress in hearts. Notably, mitochondrial ROS (mtROS) is the main resource of oxidative stress. Previous studies showed dioscin protects against pulmonary injury by inhibition of the mtROS by activating autophagy (12). Dioscin also activates NRF2 and SIRT2 signal pathway to suppress ROS in doxorubicin-induced cardiotoxicity (21). Moreover, dioscin preventing the accumulation of ROS via activating FXR/LKB1 signal in ischemia/reperfusion injury (22). Although dioscin could improve mitochondrial function in pathological condition, whether it is involved in Kreb's cycle of mitochondria remained unclear.

The Kreb's cycle is a series of complex chemical reaction, which is occurred in organisms to generate energy (23). We detected the essential enzyme activities in mitochondrial Kreb's cycle and respiratory chain, which were enhanced after diosicin administration against the infarction/hypoxia injury. The selective accumulation of the citric acid cycle intermediate succinate is a typical metabolic issue after infarction in a range of tissues, responsible for the accumulation of mitochondrial ROS production after reperfusion (23). The i*n vivo* reduction in the activities of Kreb's cycle in infarcted hearts may represent at an early maladaptive phase in the metabolic alterations after MI (24). It has been reported that high-throughput liquid chromatography-mass spectrometry (LC-MS)-based metabolomics demonstrate that dioscin could be associated with gluconeogenesis, Kreb's cycle, and butanoate metabolism (24). Consistently with the previous results, we further confirmed that dioscin is involved with Kreb's cycle. However, the insight underlying mechanism between disocin and Kreb's cycle requires further study.

Many studies showed that dioscin could, prevent infarction/hypoxia-induced apoptosis via down-regulating the *Bax* expression (25). Furthermore, dioscin is presented as a therapeutic nature product to maintain mitochondrial membrane potential (26). Our study suggested that the mitochondrial member potential was protected by dioscin administration in response to infarction/hypoxia, and further suppressed the apoptosis of cardiomyocytes.

In summary, the findings of current study indicate the cardioprotective role of dioscin against cardiac injury by suppressing the production of ROS in mitochondria, enhancing the activities of Kreb's cycle enzymes and respiratory chain complexes, and maintaining the structure and function of mitochondria. Our study could help to better understanding the mechanism underlying the dioscin treatment of infarcted heart diseases.

## Data Availability Statement

The original contributions presented in the study are included in the article/supplementary material, further inquiries can be directed to the corresponding authors.

## Ethics Statement

The animal study was reviewed and approved by Institutional Animal Care and Use Committees at the University of Nanjing Medical University (Ethical Approval Number: 1912034).

## Author Contributions

QL and HS designed the study and revised the manuscript. DL, TS, and MZ carried out the experiments and wrote the manuscript. All authors contributed to the article and approved the submitted version.

## Funding

This study was supported by the grants from the National Natural Science Foundation of China (81970414 to QL) and the Natural Science Foundation of the Jiangsu Higher Education Institutions of China (19KJA35000 to QL).

## Conflict of Interest

The authors declare that the research was conducted in the absence of any commercial or financial relationships that could be construed as a potential conflict of interest.

## Publisher's Note

All claims expressed in this article are solely those of the authors and do not necessarily represent those of their affiliated organizations, or those of the publisher, the editors and the reviewers. Any product that may be evaluated in this article, or claim that may be made by its manufacturer, is not guaranteed or endorsed by the publisher.
